# Short courses

**DOI:** 10.1155/S1463924689000027

**Published:** 1989


					G. W. Kramer et al. A simple device for the precise addition of liquids
Header file pcadder.h
/* FILE PCADDER.H
Desc: Header file for the slow-adder routines
Date: 02/12/88
Vers: 1"00
Auth: ARF/JMH
,/
#define PRIMMAX
#define PRIMOK
4O
#define PRIMERR
#define PRIMD
/* max. pulses to prime
before returning error */
5 /* min. positive readings
to confirm prime*/
/* error returned ifcan't
prime*/
1000 /* time between prime
pulses (msec.) */
#define ADDOK 5 /* min. positive readings
to confirm addn. end */
#define ADDERR -2 /* error returned on
addition timeout */
#define FLUSHNO 5 /* number ofpulses at end
to empty tubing */
#define FLUSHD 1000 /* time between flush
pulses (msec.) */
#defineTIMEMULT 1"25 /* maximum addition
time before returning
error is calculated time
* TIMEMULT */
#define MINCALIB 0"001 /* Sets the minimum drop
size at l/pulse */
#define MAXPULSE /* Sets the max pulse rate
to pulse/s */
Short courses
Loughborough University of Technology, UK, has
announced the following short courses for 1989:
Fluorescence and Luminescence Spectrometry- 26-30
June 1989. Fee 480 including residence and all meals
(450 if paid with booking form). Non-residents 405
(375).
Statistics for Analytical Chemistry- 11-14 July 1989.
Fee 385 including residence and all meals (355 if paid
with booking form). Non-residents 325 (295).
Flow Injection Analysis- 12-14 July 1989. Fee 325
including residence and all meals (300 if paid with
booking form). Non-residents 275 (250).
For further details please contact: Mrs J. E. Stirling,
Department of Chemistry, Loughborough University of Tech-
nology, Loughborough, Leics. LEll 3TU. Telephone: (0509)
222549.
HPLC Technology and Applied Chromatography
Systems are holding five HPLC Beginners Training
Courses during 1989. Each course will last three days and
will include both practical and discussion sessions. The
courses are held at The Deanwater Hotel, Woodford,
Cheshire. All purchasers of HPLC Systems from ACS
receive a complimentary place on the course.
For further information please contact: Applied Chromato-
graphy Systems, The Arsenal, Heapy Street, Macclesfield,
Cheshire SKll 7JB. Telephone: (0625)34575.
Chemserve have announced two courses for 1989:
Fluorine Spectroscopy Workshop- 10-11 April 1989.
Mass Spectrometry for Beginners- 17-18 April 1989.
Forfurther information, please contact: Chernserve, UMIST,
PO Box 88, Manchester M60 1QD. Telephone: 061 228 7700.
The University of Liverpool is holding a short course on
Modern Spectroscopic Techniques, 2-7 April 1989.
Forfurther information, please contact: DrA. Hodgson, Dept.
of Chemistry, University ofLiverpool, PO Box 147, Liverpool
L69 3BX.
Royal Society of Chemistry Residential School- 28-31
March 1989. Computer Methods in UV, vis and ir
Spectroscopy, Polytechnic of Wales.
Forfurther information, contact Ms L. Hart, RSC, 30 Russell
Square, London WC1B 5DT. Telephone: 01-631-1355.
14

				

